# Genome Sequence Analysis of *Clostridium chauvoei* Strains of European Origin and Evaluation of Typing Options for Outbreak Investigations

**DOI:** 10.3389/fmicb.2021.732106

**Published:** 2021-09-29

**Authors:** Prasad Thomas, Mostafa Y. Abdel-Glil, Inga Eichhorn, Torsten Semmler, Christiane Werckenthin, Christina Baumbach, Wybke Murmann, Anne Bodenthin-Drauschke, Pia Zimmermann, Ulrich Schotte, Domenico Galante, Durda Slavic, Martin Wagner, Lothar H. Wieler, Heinrich Neubauer, Christian Seyboldt

**Affiliations:** ^1^Institute of Bacterial Infections and Zoonoses, Friedrich-Loeffler-Institut, Jena, Germany; ^2^Department of Veterinary Medicine, Institute of Microbiology and Epizootics, Freie Universität Berlin, Berlin, Germany; ^3^Robert Koch Institute, Berlin, Germany; ^4^Lower Saxony State Office for Consumer Protection and Food Safety (LAVES), Food and Veterinary Institute Oldenburg, Oldenburg, Germany; ^5^State Office for Agriculture, Food Safety and Fisheries Mecklenburg-Western Pomerania, Rostock, Germany; ^6^Chemical and Veterinary Investigations Office, Freiburg, Germany; ^7^Landeslabor Schleswig-Holstein, Neumünster, Germany; ^8^Bavarian Health and Food Safety Authority (LGL), Laboratory of Food Microbiology, Oberschleißheim, Germany; ^9^Department A-Veterinary Medicine, Central Institute of the Bundeswehr Medical Service Kiel, Kronshagen, Germany; ^10^Istituto Zooprofilattico Sperimentale della Puglia e della Basilicata, Foggia, Italy; ^11^Animal Health Laboratory, Laboratory Services Division, University of Guelph, Guelph, ON, Canada; ^12^Unit for Food Microbiology, Institute for Food Safety, Technology and Veterinary Public Health, University for Veterinary Medicine, Vienna, Austria

**Keywords:** strain typing, *Clostridium chauvoei*, genome analysis, pangenome SNPs, CRISPR spacer-typing, cgMLST, outbreak investigation, virulence factors

## Abstract

Black quarter caused by *Clostridium (C.) chauvoei* is an important bacterial disease that affects cattle and sheep with high mortality. A comparative genomics analysis of 64 *C. chauvoei* strains, most of European origin and a few of non-European and unknown origin, was performed. The pangenome analysis showed limited new gene acquisition for the species. The accessory genome involved prophages and genomic islands, with variations in gene composition observed in a few strains. This limited accessory genome may indicate that the species replicates only in the host or that an active CRISPR/Cas system provides immunity to foreign genetic elements. All strains contained a CRISPR type I-B system and it was confirmed that the unique spacer sequences therein can be used to differentiate strains. Homologous recombination events, which may have contributed to the evolution of this pathogen, were less frequent compared to other related species from the genus. Pangenome single nucleotide polymorphism (SNP) based phylogeny and clustering indicate diverse clusters related to geographical origin. Interestingly the identified SNPs were mostly non-synonymous. The study demonstrates the possibility of the existence of polymorphic populations in one host, based on strain variability observed for strains from the same animal and strains from different animals of one outbreak. The study also demonstrates that new outbreak strains are mostly related to earlier outbreak strains from the same farm/region. This indicates the last common ancestor strain from one farm can be crucial to understand the genetic changes and epidemiology occurring at farm level. Known virulence factors for the species were highly conserved among the strains. Genetic elements involved in Nicotinamide adenine dinucleotide (NAD) precursor synthesis (via nadA, nadB, and nadC metabolic pathway) which are known as potential anti-virulence loci are completely absent in *C. chauvoei* compared to the partial inactivation in *C. septicum.* A novel core-genome MLST based typing method was compared to sequence typing based on CRISPR spacers to evaluate the usefulness of the methods for outbreak investigations.

## Introduction

Blackleg, primarily a disease of ruminants, also known as “black quarter,” “symptomatic anthrax,” “quarter evil,” “Rauschbrand,” “Geräusch,” and “charbon symptomatique” occurs worldwide. *Clostridium chauvoei*, the causative organism for blackleg, is a Gram-positive, motile, spore-producing, anaerobic bacterium. Blackleg in cattle is an endogenous infection and occurs without a wound or break of the skin (Hatheway, 1990). The majority of blackleg cases occur in young cattle due to ingestion of spores. The disease is characterized by inflammation and necrosis of skeletal and cardiac muscles, toxemia and sudden death. Sheep get infected through skin wounds after shearing, castration and tail docking (Quinn et al., 2011), and the pathogenesis mostly resembles malignant oedema (Songer, 1998). The disease has also been reported for deer, mink, and ostrich (Armstrong and Macnamee, 1950; Langford, 1970; Lublin et al., 1993). Fatal cases of human *C. chauvoei* infections were reported in recent years (Nagano et al., 2008; Weatherhead and Tweardy, 2012). There is likely an underreporting of *C. chauvoei* infection in humans and thus an underestimation of its zoonotic potential.

The pathogenesis of blackleg is not fully known and is presumed to begin with ingestion of spores by the animal while grazing followed by translocation to the bloodstream (Useh et al., 2006; Pires et al., 2017). The existence of viable vegetative cells and spores in murine and bovine macrophages has also been reported (Pires et al., 2017). This hence supports a latency phase existence in animal muscle tissue after being transported by macrophages from the intestine (Abreu et al., 2017).

Several species within the genus *Clostridium* have large plasmids and plasmid-encoded toxin genes as reported for *C. perfringens, C. sordellii*, *and C. botulinum* (Marshall et al., 2007; Couchman et al., 2015; Freedman et al., 2015). Evolution of spore formers is a complicated field of theory as they can move to a stage of inactivity and persistence. A recent study with 200 *Firmicutes* species showed that spore-forming bacteria have longer generation times and evolve more slowly. Sporulation hence significantly reduces the genome-wide spontaneous DNA mutation and protein evolutionary rates over time (Weller and Wu, 2015).

Next-generation sequencing techniques have made population genomics an available and useful tool for epidemiological studies (Joseph and Read, 2010). After the first report of whole-genome sequence data for a virulent strain from Switzerland (Falquet et al., 2013), the pathogens’ genome components and potential virulence factors were unraveled at a genomic level (Frey and Falquet, 2015). Comparative genome studies involving three complete *C. chauvoei* genomes showed limited variability of orthologous genes, provided insights into the phylogenetic position of the species, CRISPR elements, and variations in the genes regulating sporulation and germination in the genus (Thomas et al., 2017). Genome sequencing and comparative genomics involving 20 strains from a wider geographical origin revealed limited genetic variability for the pathogen (Rychener et al., 2017). A group of strains originating from Australia, New Zealand, and the United Kingdom showed remarkable differences to those coming from Europe, Africa, and America and thus indicated a different ancestral lineage (Rychener et al., 2017).

The pathogen harbors *Clostridium chauvoei* toxin A (*CctA*) which belongs to the leucocidin superfamily of bacterial toxins. CctA is considered the major virulence factor and a potent protective antigen target for vaccines against blackleg (Frey et al., 2012; Frey and Falquet, 2015; Rychener et al., 2017; Thomas et al., 2017; Gupta et al., 2020). The toxin was also found to be well conserved in different *C. chauvoei* strains except for one amino acid substitution in two strains and few silent mutations (Rychener et al., 2017). Sialidases and hyaluronidases are other virulence factors of *C. chauvoei* (Useh et al., 2003). Sialidase (*nanA*) and Hyaluronidases (*nagH* and *nagJ*) exhibited genetic variability among the strains coming from Australia, New Zealand, and the United Kingdom as compared to strains originating from other regions (Rychener et al., 2017).

The current study was designed to investigate the population diversity of *C. chauvoei* among 64 stains. The strains were mostly isolated from blackleg outbreak cases from Europe, the majority from Germany, Austria and a few from non-European and unknown origin. Some of these were strains isolated from the same animal, from the same outbreak, from the same farm, and in some cases with temporal intervals, which provided the opportunity to understand the evolution of the pathogen. Currently there are no comprehensive data on the population structure and diversity of this important animal pathogen in detail within Europe. Genotypes are also not known for the species, and no typing scheme based on sequence data is currently available except for the CRISPR spacer based strain differentiation described by Rychener et al. (2017). Hence, the current study was designed to investigate the genetic diversity, population structure and evolution of *C. chauvoei* and to propose a molecular typing tool. In this study, whole-genome sequencing and comparative genomics studies were applied to analyze the core, accessory and pangenome, to infer a phylogeny as well as to develop typing methods.

## Materials and Methods

### Bacterial Strains, DNA Extraction, and Sequencing

**FIGURE 1:**
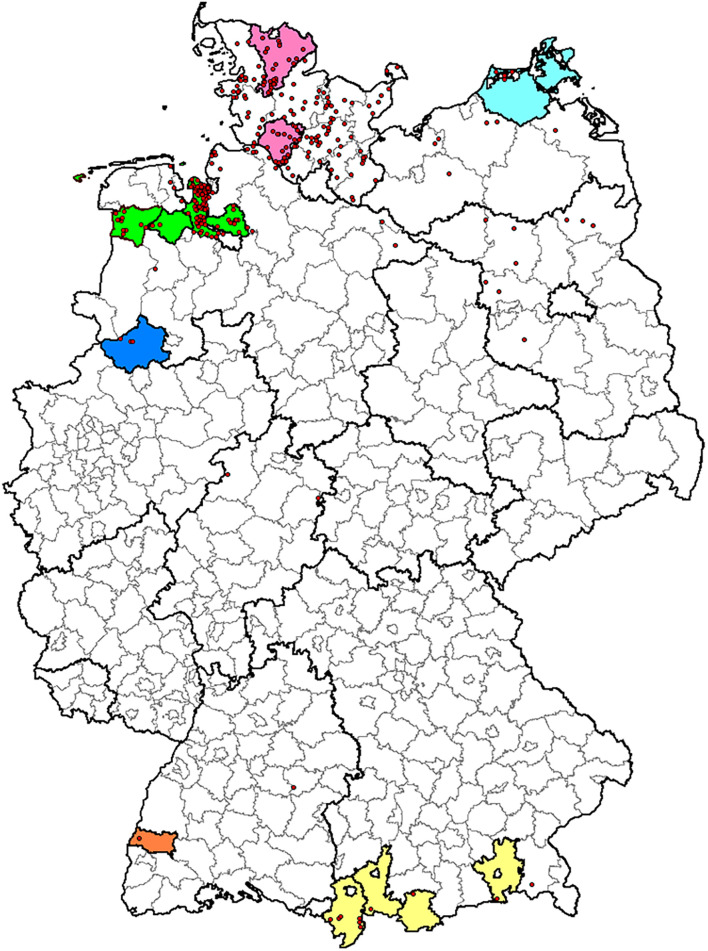
Geographical map of blackleg outbreaks in Germany from 1995 to 2016. Map depicting the origin of the German *C. chauvoei* isolates used in this study (colored areas) and reported outbreaks (red dots) from 01.01.1995 to 31.12.2016 (*n* = 334). Borders of federal states and administrative districts are delineated. Color coding: Lower Saxony: green, North Rhine-Westphalia: blue, Schleswig-Holstein: pink, Mecklenburg-Western Pomerania: aqua, Baden-Württemberg: orange and Bavaria: yellow.

Bacterial strains used in the study were maintained in the culture collection of the Institute of Bacterial Infections and Zoonoses (IBIZ), Friedrich-Loeffler-Institut (FLI), Jena, Germany. *C. chauvoei* strains (60 strains) were mainly of European origin representing Germany (38 strains), Austria (ten strains), Switzerland (two strains), Italy (one strain), and few strains of unknown origins (nine strains). Within Germany, blackleg outbreaks are reported from six different states and the strains involved in the study were representative from all these reported states (Annual Animal Health Report, 2017^1^). Schematic representation of blackleg outbreaks in Germany from 1995 to 2016 is shown in a geographical map in Figure 1. One strain was from Canada and another strain (NCTC 08361) from a sheep in South Africa. In addition, four previously published strains of *C. chauvoei* (genome sequence/reads) were included in the analysis (Falquet et al., 2013; Thomas et al., 2017). Details of strains involved in the current study with respect to epidemiological data (host, year, and country of origin) and Sequence Read Archive (SRA)/Genome assembly accession numbers are provided in Supplementary Table 1. These strains were mostly isolated from various tissues such as muscles, spleen, and liver following blackleg outbreaks. All strains were cultured on sheep blood agar plates (Yeast Extract Cysteine Medium with Sheep Blood (Beerens Formulation) Thermo Scientific, Oxoid, Germany) at 37°C for 24–48 h under anaerobic conditions. The culture material scraped off from one to two agar plates was used for DNA extraction. The genomic DNA was isolated using DNeasy Blood and Tissue Kit (Qiagen, Germany) with slight modification, as 40U of achromopeptidase (Frey et al., 2012) was included in the enzymatic buffer along with lysozyme. A PCR for species confirmation was based on published primers specific region for 16–23 S rDNA spacer regions for differentiating *C. chauvoei* and *C. septicum* (Sasaki et al., 2000). Library preparation from genomic DNA was carried out using Nextera^TM^ (Illumina, Netherlands) library preparation method. Genome sequencing was carried out using MiSeq^TM^ System (Illumina, United States) paired-end sequencing technology (2 × 300bp) at the Institute of Microbiology and Epizootics (IMT), Freie Universität Berlin, Berlin.

### Genome Assembly and Annotation

Paired-end Illumina reads were first checked for quality using the FastQC version 0.11.6^2^). Kraken2 was then used for taxonomic classification (Wood and Salzberg, 2014). For the assembly, we used Shovill version 1.0.4^3^ using the option “-trim” enabling adapter and quality trimming using Trimmomatic version 0.39 (Bolger et al., 2014). The two complete genome sequences (DSM 7528^T^ and 12S0467) previously published (Thomas et al., 2017) based on PacBio assembly, were downloaded and polished for four rounds with Illumina reads using Pilon tool version 1.22 (Walker et al., 2014). Assembly statistics were generated using QUAST (Gurevich et al., 2013). Genome annotations were carried out using Prokka version 1.11 (Seemann, 2014). The presence of previously reported plasmid (4.1Kb; NZ_CP018631.1) was searched among the strain assembly files using ABRicate version 1.0.1^4^.

### Repeat Elements and Prophages

CRISPR elements and spacers were predicted using the CRISPRDetect program (Biswas et al., 2016) followed by CRISPR array visualization using CRISPRStudio (Dion et al., 2018). When CRISPR regions were represented in more than one contig (six strains), the orientation and directions were assigned as per DSM 7538^T^ strain. Prophage elements and genomic islands were predicted using Prophage Hunter web tool (Song et al., 2019) and IslandViewer 4 (Bertelli et al., 2017), respectively. BLAST atlas of all the genomes with locations of prophages and genomic islands was created using BLAST Ring Image Generator (BRIG) (Alikhan et al., 2011).

### Pangenome Analysis

Pangenome analysis was carried out with Panaroo pipeline version 1.2.7 (–clean-mode strict; –remove-invalid-genes) (Tonkin-Hill et al., 2020) using the Prokka (Seemann, 2014) annotated genomes. Curves for the core and pangenome were calculated and plotted using PanGP version 1.0.1 (Zhao et al., 2014). New gene discovery was plotted as a function of the number of genomes added sequentially using a distance guide algorithm with 100 replicates and 3000 permutations of genome order. PanGP uses power-law regression (y = A^xB^ + C) to model the pangenomes generated from all permutations, where y is the total number of gene families in the pangenome, x is the number of genomes considered, A, B, and C are fitting parameters. When 0 < B < 1, the pangenome should be considered open. The exponential curve fit model, y = Ae^Bx^ + C was used to fit the core genome. Here, y denotes core genome size, x denotes the number of genomes; A, B and C are fitting parameters. The curve for new gene discovery represents the least-squares fit for the function y = A^xB^, where y denotes the new genes, x denotes the number of genomes and A and B are the fitting parameters.

### Single Nucleotide Polymorphism Analysis and Recombination

Single nucleotide polymorphism analysis was carried out using Snippy version 3.2^5^. The pipeline maps read-pairs to reference strain (DSM 7528^T^) using Burrows-Wheeler Aligner version 0.7.12 (Li and Durbin, 2009). Average read depths were calculated with SAMtools version 1.2 (Li et al., 2009) and SNPs were identified using FreeBayes version 0.9.21 (Garrison and Marth, 2012) with a minimum depth of 5x and a minimum variant allele proportion of 0.9. Snippy was then used to pool all identified SNP positions (excluding indels) called in at least one isolate, and a multiple sequence alignment of core SNPs was generated. Recombination analysis of the strains based on the core genome was carried out using Gubbins version 2.2.1 (Croucher et al., 2015) with default parameters. Pairwise SNP differences between groups of strains based on geographical origin were calculated and summarized for strain groups using pairwise_snp_differences^6^. The BCFtools^7^ was used to extract strain-specific and group-specific SNPs from outbreak strains that were recovered from the same animal or different animals on the same farm. Protein coding genes that harbor any of the identified SNPs were extracted and functional annotation was carried out using OmicsBox version 1.4.11 (Götz et al., 2008; BioBam Bioinformatics., 2019).

### Phylogeny

Pangenome SNP analysis independent of multiple alignments and a reference genome was carried out using kSNP3.0 (Gardner et al., 2015). kSNP3 identifies SNPs based on unique stretches of nucleotides present in all genomes having SNPs in their middle position. The optimal size of the nucleotide regions flanking the SNPs (kmer) was identified using the program Kchooser available with the package. The SNP matrix file (SNPs_all_matrix.fasta) generated by the tool was used to produce a parsimony tree, which is a consensus of up to 100 equally parsimonious trees. The phylogenetic tree was visualized using Interactive Tree Of Life (iTOL) version 4 (Letunic and Bork, 2019).

### Core Genome Multilocus Sequence Typing

A core genome-based multilocus sequence typing (cgMLST) analysis was performed for *C. chauvoei* using Ridom SeqSphere version 7.1.0 (Junemann et al., 2013). The tool “cgMLST Target Definer” extracted genes from the reference genome (DSM 7528^T^; NZ_CP018624.1) and compared these genes through BLAST (Altschul et al., 1990) against multiple query genome sequences with gene identity and query coverage set to 90 and 100%, respectively. The 63 assembled genomes in this study were used as query genomes. BLAST (blastn) options included match reward 1, word size 11, gap open costs 5, mismatch penalty −1, gap extension cost 2. The analysis identified cgMLST targets and accessory targets. SeqSphere software assigned alleles for each gene and generated an allelic profile for all strains. Allelic profile data were used to generate minimum spanning trees (MST) using the parameter ‘‘pairwise ignore missing values’’ during distance calculation. The developed *C. chauvoei* cgMLST scheme is publicly available at PubMLST.org website^8^ (Jolley et al., 2018).

### Virulence Factors and Antivirulence Loci

Variations in primary virulence factors were studied based on sequence searches using ABRicate version 1.0.1^9^ with custom sequence databases followed by alignment of individual genes from all strains. Antivirulence loci (AVL) are genetic elements present in genomes of an ancestor but absent or inactive in the pathogen since AVL expression is detrimental to the expression of some virulence phenotype. Besides, variation concerning primary virulence factors was studied based on protein alignments of individual genes created using all strains followed by visualization in Geneious Prime 2019 (Kearse et al., 2012). The presence or absence of antivirulence loci in *C. chauvoei* genome sequences was determined based on ABRicate [32] searches using identified homolog AVL loci (nadA, nadB, and nadC; 3.1Kb) from *Clostridium septicum* DSM 7534^T^ strain (NZ_CP023671). Nicotinate and nicotinamide metabolism pathways for *C. chauvoei* and *C. septicum* were accessed through KEGG database (Kanehisa et al., 2017).

## Results

### Strain Assembly and Annotation

Taxonomic classification of the sequence reads revealed 98% read homology for all strains with the NCBI *Clostridium* databases except one strain (strain 16S0574) whereas only 92% of the reads have matched. Further analysis of this strain showed read contamination with exogenous genetic material; hence we first mapped the sequence data of this strain to reference genomes of *C. chauvoei* previously described (Thomas et al., 2017), and only the mapped reads were filtered out and used for subsequent analysis. The GC content averaged 27.9% for all strains. The *de novo* genome assembly and annotation of the strains reflected similar genome sizes and number of encoded proteins respectively. The genome size of the assembled genomes varied between 2.76 and 2.79 Mb for all strains, with N50 values between 63 and 90Kb and a total contig number between 63 and 81 contigs per strain (Supplementary Table 2). Genome annotation revealed 2560 to 2581 protein coding genes among the strains (Supplementary Table 2).

### CRISPR Elements and Prophages

The genomes harbored two CRISPR repeat sites (repeat region 1 and 2) separated by a protein coding gene except for one strain of unknown origin (S0132-09) where the CRISPR repeat region was continuous. The strains showed variations in the composition of CRISPR spacer sequences among the isolates. Variations were observable at the CRISPR site region which was close to the *Cas* proteins. The total number of CRISPR spacers among all the strains was 2,608 (1,988 for repeat region 1 and 620 for repeat region 2, respectively) whereas the number of unique spacers identified was 45 (35 for repeat region 1 and 10 for repeat region 2, respectively). The maximum and minimum number of spacers ranged from 35 to 17 and 10 to 8 for repeat regions 1 and 2, respectively. Most of the strains from Europe and the type strain had 43 spacers (33 and 10 spacers at repeat regions 1 and 2, respectively). The number of spacers identified for each strain at repeat regions, positions of spacers in form of an array is detailed in Supplementary Table 3. Two strains (12S0470; spacer 44 and S0132-09; spacer 45) each had an additional unique spacer that was not present in the type strain (Supplementary Table 3). Recent studies have pointed out the applicability of CRISPR spacers for differentiation of strains of *C. chauvoei* (Rychener et al., 2017). CRISPR spacers for all 64 strains were visualized as an array and were representing 18 unique patterns. The CRISPR spacers, array pattern, strain name, and region wise and country information are represented in Supplementary Figure 1. The array for spacers showed marked variations with respect to strains from Austria, Switzerland, 15S0023 (South Africa), strains from North Rhine-Westphalia, Bavaria and few strains from Lower Saxony and of unknown origin. Prophage Hunter tool predicted prophages categorized as active/ambiguous and with a prediction score above 0.5 were considered (Song et al., 2019). Four prophages and nine genomic islands were predicted for *C. chauvoei* strain DSM 7528^T^, respectively. The sizes and protein coding genes encoded by respective prophages and genomic islands are shown in Supplementary Table 4. BRIG blast atlas showing sequence variability among strains and with respect to the prophages and genomic islands are depicted in Supplementary Figure 2.

### Core and Accessory Genome

**FIGURE 2:**
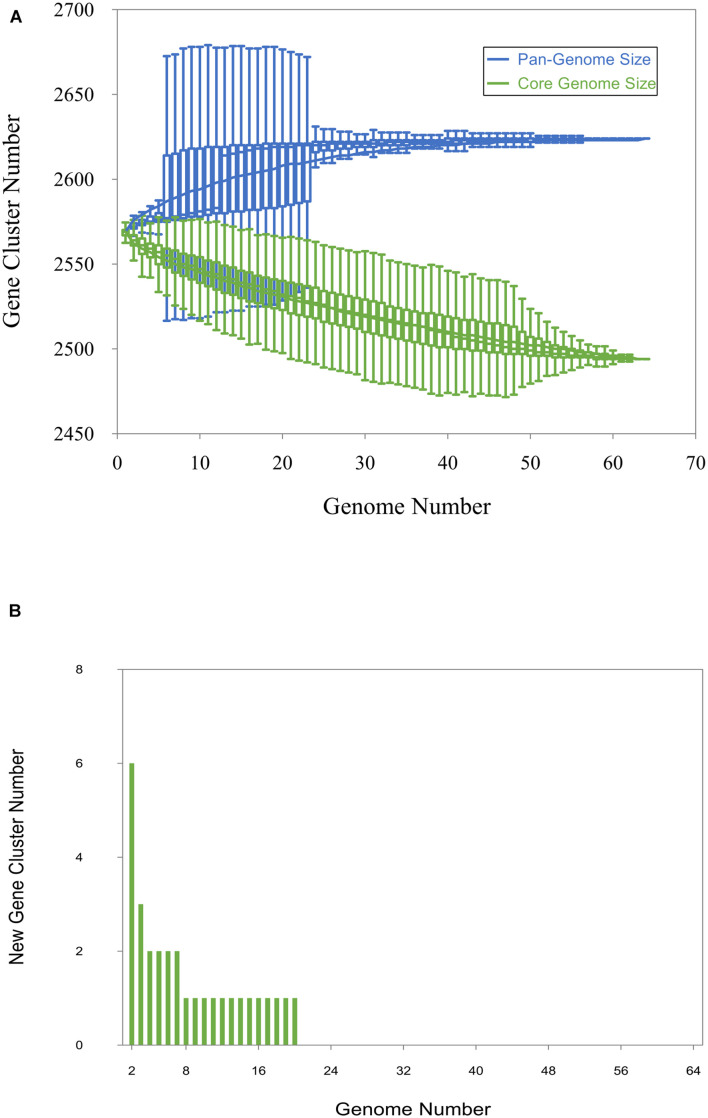
Pan-genome, core genome and new gene plot. **(A)** The pan-genome plot (blue) and core genome plot (green). For the pan-genome and the core genome analysis, the number of genes is plotted as a function of the number (n) of strains sequentially added. The curve for the pangenome represents the least-squares fit for the function y = A^xB^ + C with the best fit obtained with a correlation *r*^2^ = 0.986 for A = 140.68, B = 0.08, C = 2425.06. The curve for the core genome represents the least-squares fit for the function y = A^eBx^ + C with the best fit obtained with a correlation *r*^2^ = 0.997 for A = 90.4, B = −0.02, C = 2,475.36. **(B)** The new gene plot. The curve for new genes represents the least-squares fit for the function y = Ax^B^ with the best fit obtained with a correlation *r*^2^ = 0.835 for A = 39.854, B = –1.37.

Panaroo predicted 2,494 core genes strictly present in the 64 investigated strains comprising more than 95% of the pangenome of the strains (total 2,624 pangenes), while the accessory genome comprised less than 5% (148 out of 2,624 genes). The curve for the pangenome represents the least-squares fit for the function y = Ax^B^ + C with the best fit obtained with a correlation *r*^2^ = 0.986 for A = 140.68, B = 0.08, C = 2425.06 (Figure 2A). The extrapolated *C. chauvoei* pangenome size expected after the number of genomes increases to 100 was 2,628. The fitting parameter B value was 0.08 i.e., between 0 and 1. The number of core genes after the addition of each new genome was plotted as a function of the number of genomes to create a core genome plot. The curve for the core genome represents the least-squares fit for the function y = Ae^Bx^ + C with the best fit obtained with a correlation *r*^2^ = 0.997 for A = 90.4, B = −0.02, C = 2,475.36 (Figure 2A). The extrapolated *C. chauvoei* core genome size for 100 genomes was estimated as 2,487 genes. The curve for new genes represents the least-squares fit for the function y = Ax^B^ with the best fit obtained with a correlation *r*^2^ = 0.835 for A = 39.854, B = −1.37 (Figure 2B). The extrapolated number of new genes expected after the number of genomes increases to 100 was zero indicating a closed pangenome structure. Accessory genes were majorly comprised of genes related to prophages, mobile genetic elements and insertional sequences. The variations attributed by the strains even involving different geographical locations were limited as there was no clear pattern of accessory genes sharing among the strains.

### Pairwise Single Nucleotide Polymorphism Variations and Recombination

A total of 1,677 core genome SNPs was identified among all strains based on the sequence read mapping to the DSM 7528^T^ reference genome by Snippy analysis. Core SNP density showed an almost even distribution across genomes and ranged from 31 to 81 SNPs within every 100Kb genomic regions. Pairwise SNP variations ranged from 0 to a maximum of 331 SNPs among any pair of isolates. Pairwise SNP differences among strains are depicted in Supplementary Table 5. Pairwise SNP difference analysis within groups based on geographical origin showed a high median pairwise SNP difference value of 89 SNPs (10 strains) for Austria as compared to 41 SNPs (38 strains) from Germany (Supplementary Figure 3A). Similarly, the strains from the mountain pastures (Bavaria, Austria and Switzerland) showed a higher pairwise SNP difference value of 77 (17 strains) as compared to 38 (34 strains) from plain regions (Lower Saxony, North Rhine-Westphalia, Mecklenburg-Western Pomerania, and Schleswig-Holstein) (Supplementary Figure 3B). This is indicative of higher strain diversity in seasonal mountain pastures as compared to other regions.

The dataset investigated herein included strains obtained from six outbreaks in Germany. Each of these outbreaks was represented by two to three *C. chauvoei* strains. Though phylogenetic analysis revealed that the outbreak strains were closely related, the pairwise SNP distances (Supplementary Table 5) between these strains revealed a genetic distance between 11 to 112 SNPs. These strains were however isolated either from the same animal (one outbreak) or from two different animals on the same farm in the event of the outbreak (five outbreaks). To understand the genetic variations associated with these outbreak strains, we parsed the Snippy output files using BCFtools and extracted SNP variations among the outbreak strains. To define SNPs exclusively associated with outbreak strains, closely related strains that were not isolated during the outbreak were not included. For example, we excluded strain S0121-09 that was isolated in 2009 but was phylogenetically grouped with the 2010 outbreak strains (S0121-10 and S0122-10) from North Rhine-Westphalia. Similarly, we excluded strain S0260-09 isolated in 2009 but grouped with the 2012 outbreak strains (12S0468 and 12S0467) in Lower Saxony. The number of SNPs identified from the same host and outbreak strains is shown in Table 1. In total, 240 strain-specific SNPs were identified among the same animal and outbreak strains, 194 SNPs belonged to protein-coding genes and were predominately represented by non-synonymous (NS) substitutions indicating diversifying selection occurring inside the host. The number of SNP variations uniquely represented by strains recovered in the same animal and from five outbreaks is shown in Supplementary Table 6. SNPs in different positions for the same protein-coding genes observed for the outbreak strains were coding for HTH-type transcriptional regulator CymR and Nitrate reductase (three SNPs) and Sensor histidine kinase RcsC, Putrescine transporter PotE, putative M18 family aminopeptidase 1, Magnesium transporter MgtE, Putative competence-damage inducible protein, Flagellar biosynthesis protein FlhA, Leucine–tRNA ligase, UDP-3-O-(3-hydroxymyristoyl) glucosamine N-acyltransferase and for three proteins coding genes predicted as hypothetical proteins (two SNPs). Based on Blast2GO, these genes were involved in various biological process and predominantly for cellular macromolecule biosynthetic process (GO:0009059, GO:0044260, GO:0044249) (Supplementary Figure 4). Based on KEGG pathway analysis, 21 CDS were involved in purine metabolism followed by thiamine metabolism (13 CDS), amino sugar and nucleotide sugar metabolism (7 CDS).

**TABLE 1:** Single nucleotide polymorphism (SNP) variations of strains from one outbreak or animal.

Groups	Strain involved	SNPs unique			Total SNPs
**Same animal strains**					
Group_1	11S0315, 11S0316 & 12S0471	11S0315	11S0316	12S0471	
		16	35	11	62
**Outbreak strains**					
Group_2	S0021-10 & S0022-10	S0021-10	S0022-10		
		3	28		31
Group_3	S0040-08 & S0041-08	S0040-08	S0041-08		
		13	55		68
Group_4	13S0851 & 13S0854	13S0851	13S0854		
		5	7		12
Group_5	BS79-01 & BS80-01	BS79-01	BS80-01		
		10	21		31
Group_6	12S0468 & 12S0467	12S0468	12S0467		
		5	31		36

*Number of SNPs identified from same animal and outbreak strains (recovered from different animals during one outbreak). The table shows the strains isolated from one animal (Group_1) and outbreak strains (group 2 to 6), strain designation, number of unique SNPs predicted for each strain and the total SNP numbers within same host/outbreak strains are shown.*

Recombination analysis of the strains under study showed limited recombination events based on Gubbins predictions. Eight recombinant regions were predicted involving a minimum number of 4 to a maximum of 14 SNPs. Among eight, three regions involved protein coding genes representing capsular polysaccharide biosynthesis protein (10 SNPs), flagellin C (14 SNPs) and metal ABC transporter permease (6 SNPs).

### Phylogeny and Clustering

**FIGURE 3:**
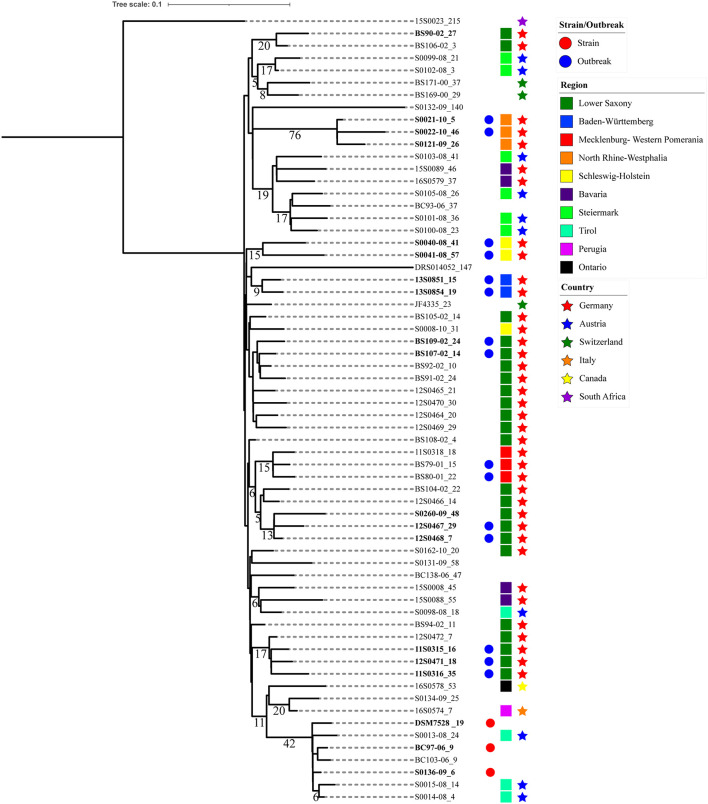
Phylogenetic tree based on *Clostridium chauvoei* pan-genome SNPs. Phylogenetic tree based on the pan-genome SNPs with branch lengths expressed in terms of changes per number of SNPs and the node labels showing the number of SNP alleles (>5) that are specifically present in all descendants of that node. The tree represents the strain/outbreak/farm, region and country level relationship among the strains whereas empty spaces represent unknown information. The strain specific allele counts are indicated after an underscore symbol (_) connected with the strain name. The clusters observed in the tree were mostly showing geographical relationship. The SNPs shared by a cluster had a maximum value of 76 (three strains from North Rhine-Westphalia).

Pangenome phylogenetic analysis was carried out using kSNP3. The optimal kmer value was 21. A total of 2,389 SNPs were identified in the 64 *C. chauvoei* genomes, of which core SNPs were 2,125 and non-core SNPs were 264. Among the SNPs occurring in protein-coding genes, 1,451 corresponds to non-synonymous (NS) substitutions and 178 were synonymous (S) type resulting in a high NS/S ratio of 8.151 indicating diversifying selection. A total of 45 SNPs were homoplastic which corresponds to SNPs shared by groups of genomes that are not clustered in the consensus maximum parsimony tree used for clustering the strains. The phylogenetic tree indicating the clusters with branch lengths expressed in terms of changes per number of SNPs and node labels showing shared SNPs (>5) among strains are shown in Figure 3.

The parsimony tree based on the pangenome SNPs indicated clusters defined based on the presence of SNPs shared. Three strains represented type strain ATCC 100092 (S0136-09, BC97-06 and DSM 7528^T^) received from various sources showed relatedness with respect to shared SNPs. As mentioned earlier, strains recovered from the same farm or animal/outbreak showed high genetic relatedness. Also strains originating from the same farm or region but in longitudinal time gaps of one (S0121-09, S0021-10 and S0022-10), two (12S0467, 12S0468 and S0260-09), and 4 years (BS79-01, BS80-01 and 11S0318) showed striking relatedness. This indicates persistent lineages can be involved in repeated outbreaks in the same farm (Figure 3). Exceptions were also observed when one strain from Lower Saxony (12S0464) isolated a year before (2010) was not forming a cluster with three strains isolated from the same farm a year later (11S0315, 11S0316) and one isolated from the same animal (12S0471). Similarly, strains involved in the same cluster were originating from two farms from the Lower Saxony region (BS90-02 and BS106-02); indicating diverse genotypes can be prevalent on farm/region levels.

Clusters were mostly based on the strains’ geographical origins. The SNPs shared by a cluster had a maximum number of 76 (three strains from North Rhine-Westphalia). Shared SNPs identified in the inner nodes of the clusters ranged from 0 to 42. One exceptional cluster with divergent strains was observed where the type strain (DSM 7528^T^) which was isolated before 1950 was found to be genetically related to the three strains from Austria (S0013-08, S0014-08 and S0014-08) isolated in 2008. The same cluster also involved a Canadian strain (16S0578) and was showing more relatedness to a strain of unknown origin (S0134-09). Strains from Germany were also sharing relatedness with strains from Austria. These strains were from Bavaria which shares geographical closeness to Austria. Similarly, few strains from Austria clustered with strains from Switzerland and indicate geographical proximity. Other clusters mostly involved strains from different regions of Germany viz., Lower Saxony, Lower Saxony and Mecklenburg-Western Pomerania, North Rhine-Westphalia, Schleswig-Holstein and Baden-Württemberg. Strain-specific SNPs ranged from 1 to 207. The strains harboring specific SNPs above 100 were S0132-08 (unknown origin), 1520023 (South Africa) and DRS0104052 (unknown origin) (Figure 3).

### Virulence Factors

The genome sequence analysis of strain JF4335 and previous studies have characterized primary virulence factors of *C. chauvoei* (Frey and Falquet, 2015). The identified virulence factors included *C. chauvoei* toxin A (CctA), hemolysins, sialidases, hyaluronidases and internalin A proteins (Frey and Falquet, 2015). Most of the known primary virulence factors for *C. chauvoei* showed limited variations at the amino acid level. The CctA belongs to the leucocidin superfamily of bacterial toxins and is considered the major virulence factor of the pathogen (Frey et al., 2012). The CctA showed high conservation at the amino acid level among all strains under study. Sialidases and hyaluronidases are enzymes hydrolyzing the glycoside linkage between sialic acid molecules and break hyaluronate (a carbohydrate polymer that is part of the extracellular matrix), respectively. Both virulence factors are thought to be involved in the rapid spread of *C. chauvoei* through tissues (Frey and Falquet, 2015; Abreu et al., 2017). NanA sialidase showed one amino acid substitution for strain 15S0023 (South African strain) and another one for two strains originating from Switzerland (BS169-00, BS171-00). Hyaluronidase (nagH) showed multiple deletions of amino acids for strain S0131-09 and three single substitutions were observed for three strains. Except for CctA, the role of other hemolysins in blackleg pathogenesis is unclear. There was also no variation observed for the hemolysin III and hemolysin XhlA. The amino acid substitutions and deletions identified in the virulence factors and respective positions are depicted in Table 2.

**TABLE 2:** Variations for primary virulence factors.

Primary virulence factors				
Virulence factor	Length	Protein Variation		
	(cds)	Substitution/Deletion	Number of Isolates	Strain Designation
Clostridium chauvoei toxin A (CctA)	318	None		
Hemolysin III	223	None		
Hemolysin XhlA	78	None		
Sialidase NanA	1300	Pro to Ala (1051)	2	BS169-02, BS171-02
		Ala to Asp (69)	1	1520023
Nag H	1886	64 aa deletion (1882 to1886)	1	S0131-09
		Phe to Leu (345)	1	15S0023
		Val to Gly (1439)	1	BC93-06
		Ala to Asp (1480)	1	DRS014052
Glycosyl hydrolase family 20	1735	Ala to Ser (1118)	1	15S0023
		Arg to Ser (168)	1	S0098-10
		Tryp to Ser (1040)	7	DSM 7528^T^, BC103-06, BC97-06, S0013-08, S0014-08, S0015-08, S0136-09
Hyaluronidase I (Nag I)	1321	Asp acid to Tyr (381)	1	S0013-08
		Leuc to Val (1109)	1	S0041-10
Internalin A (InlA1)	481	Stop codon (333)	3	S0134-09, 16S0578, 16S0574
Internalin A (InlA2)	447	Ser to Tyr (84)	1	15S0023
Internalin A (InlA3)	396	None		
Microbial collagenase (Col A)	983	None		

*The table depicts the identified virulence factors, coding sequence (cds) lengths, protein variations (either substitution or deletion), number of isolates involved and strain designation respectively.*

Antivirulence loci (AVL) are described as genes whose expression is incompatible with the pathogenic lifestyle. Earlier studies for *Shigella* have described two genes *nadA* and *nadB* of *Shigella* as antivirulence genes (Prunier et al., 2007). There was a notable absence of antivirulence loci encoding *nadA* (quinolinate synthase [EC:2.5.1.72]), *nadB* (L-aspartate oxidase [EC:1.4.3.16]), and *nadC* (nicotinate-nucleotide pyrophosphorylase (carboxylating) [EC:2.4.2.19]) genes in *C. chauvoei* in comparison to *C. septicum*. Both genes coding enzymes are required for the conversion of L-aspartate to quinolinate, a precursor to NAD synthesis. In contrast, all the four strains of *C. septicum* genomes (strains DSM 7534 (NZ_CP023671/NZ_CP023672.1), VAT12 (NZ_CP034358), P1044 (NZ_CABMIZ000000000.1) and MGYG-HGUT-02373 (NZ_FLTT00000000.1) were predicted for an intact coding sequence for *nadA* (100% consensus at nucleotide and protein levels) whereas both *nadB* and *nadC* coding sequences were disrupted by stop codons.

### Core Genome Multilocus Sequence Typing

**FIGURE 4:**
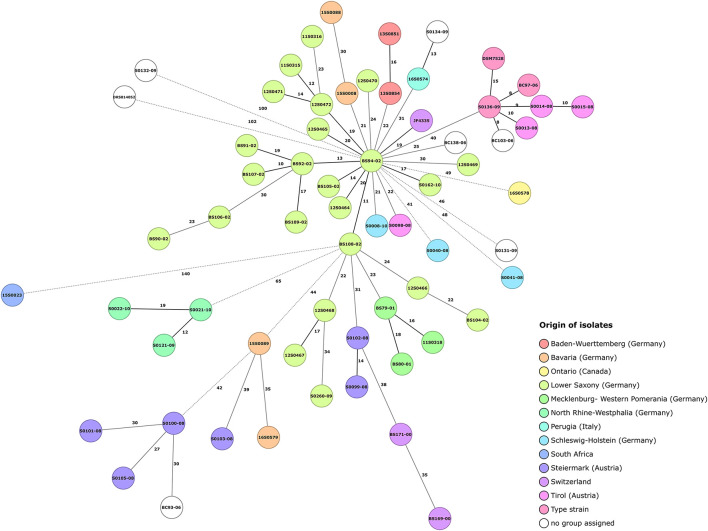
Core genome MLST minimum spanning tree for 64 *Clostridium chauvoei* isolates. *Clostridium chauvoei* cgMLST scheme employed 2,223 core genes for assessing the genetic diversity among 64 isolates. SNP distances are indicated on the branches. Allelic distances are indicated on the branches where less than 20 allelic differences, between 20 and 40 allelic differences and more than 40 allelic differences are denoted with black solid, gray solid and dashed lines, respectively. The number of different alleles is given on the connecting lines. Color grouping are based on origin of isolates.

The *C. chauvoei* cgMLST scheme consisted of 2,223 core genes for assessing the genetic diversity among isolates. The minimum spanning tree for the strains highlighting the origin information of isolates is shown in Figure 4. Similar to kSNP3 clustering, the three isolates representing the type strain (ATCC 100092) formed a group with three strains form Austria and another strain of unknown origin in MLST tree as well. This may further indicate the possibility of the type strain being initially isolated from Austria (Tyrol region) as they also represent similar CRISPR spacer patterns and share similar genetic variations for one virulence factor (Table 2). The limited genetic variations observed among the strains may be due to laboratory passages/experiments incurred SNPs and/or biases from different sequencing technologies applied in the study. The tree shows that the SNP distance for strains outside Europe is greater when compared to strains within Germany. Within Germany, the strains from North Rhine-Westphalia are the most divergent strains. Interestingly, the core genome MLST phylogeny showed collinearity with most of the outliner CRISPR arrays.

## Discussion

The study aimed to determine the population diversity of *C. chauvoei*, an important bacterial pathogen of the genus *Clostridium*, a study with 64 strains was conducted. Of these 64 strains, 38 strains were recovered from cattle blackleg outbreaks in Germany. In Germany the number of outbreaks in cattle reported from 1950 to present shows a decreasing trend averaging around 64 cases per year from 1950 to 1980 and 22 from 1980 to 2010. The decreasing trend was also observed in the subsequent years (Annual Animal Health Report, 2017).

Genomes were mostly conserved for genome size, GC content and encoded proteins (Supplementary Table 2). All the genomes in the current study carried a plasmid of 4Kb and a CRISPR locus similar to the CRISPR subtype I-B system reported earlier (Rychener et al., 2017; Thomas et al., 2017). The usefulness of the unique CRISPR spacer elements for strain typing was already demonstrated for the differentiation of *C. chauvoei* (Rychener et al., 2017) and *C. difficile* (Andersen et al., 2016). A study which was conducted with 20 *C. chauvoei* strains from a broad geographical distribution, showed some diversity and the presence of 187 unique characteristic CRISPR spacer elements. A clear clustering and very different spacer sequences for the strains from Europe/Africa/America with the strains from Australia/New Zealand/United Kingdom was reported (Rychener et al., 2017). However, in the study reported here, only 45 CRISPR spacer elements were found, indicative of less diverse strains involved. Indeed, all investigated strains belonged to the Europe/Africa/America cluster. Most of the strains showed a spacer pattern similar to that observed for the reference DSM 7528^T^ strain (Supplementary Figure 1 and Supplementary Table 3). Previous studies on *C. chauvoei* genome sequence data also reported the presence of prophages and genomic islands (Frey and Falquet, 2015; Rychener et al., 2017). The current study showed similar phage/genomic island composition amongst most strains whereas few strains showed variations with regard to its composition. This indicates that the species genomes may not be completely immune to any new phage types, but the role of phages in the evolution of this species seems to be limited as compared to other related species within the genus *Clostridium* (Knight et al., 2015).

Pangenome analysis revealed a very limited acquisition of new genes in *C. chauvoei*, with the curve depicting the expansion of the pangenome almost reaching a plateau. These results are consistent with the definition of a closed pangenome (Figure 2). This shows the low potential of *C. chauvoei* genomes for acquiring or losing large unique foreign elements. Pangenome size depends on the gene gain and loss events occurring in bacteria. Gene gain happens when the bacteria live in a very diverse environment and hence genetic exchange with diverse bacterial species is possible (Rouli et al., 2015). However, there are also species genomes with closed pangenomes such as *Yersinia pestis* and *Bacillus anthracis* known to multiply only inside the host and hence have very little chance for acquiring new genes (Rouli et al., 2015). The *C. chauvoei* core genes represented 95% of the pangenome indicative of a very limited accessory genome. In contrast, the percentage of core genes among pangenome was identified to be 11% as in the case of *C. botulinum* (Snipen et al., 2009; Rouli et al., 2015) and 12.6% in the case of *C. perfringens* (Kiu et al., 2017). One potential reason for the high core genome proportion is that the species is undergoing replication only at highly isolated places such as inside host tissues so that the chance of acquiring foreign genetic material is limited. Another probable reason could be that the species has undergone evolution of genome reduction reported in bacterial pathogens with intracellular existence where a functional reduction especially of genes involved in transcription and amino acid metabolism is detected (Rouli et al., 2015). This has been reported in an earlier study based on genome sequence data for *C. chauvoei* strain JF4335, revealing the absence of several genes involved in amino acid metabolism (Frey and Falquet, 2015). The genome size of *C. chauvoei* was remarkably small (2.8-Mb) compared to the genome of the closely related species *C. septium* (∼3.3-Mb) (Benamar et al., 2016). A pronounced loss of chromosomal genes has also been described in *C. perfringens* in phylogenetically related strains, associated with specialization to specific lifestyles (foodborne diseases) (Abdel-Glil et al., 2021). The evidence of functional reduction and intracellular existence has not been yet proven for this pathogen, even though spores and vegetative cells have been recently shown to survive in bovine macrophages (Pires et al., 2017). On the other hand, *C. chauvoei* causes massive tissue destruction with release of cellular contents. Homologous recombination in bacteria plays an important role in the evolution and often the hot spots for recombination involve bacterial virulence factors (Yahara et al., 2016). The recombination event predictions for the strains in the current study were found to be limited.

Pairwise SNP difference analysis and median pairwise SNP difference values among strains belonging to Germany and Austria showed Austrian strains to be highly variable when compared to strains originating from Germany (Supplementary Figure 3). A recent study to identify the prevalence of blackleg in Styria, Austria, involved 266 confirmed cases (18%) of 1,448 suspected cases. High prevalence was reported for North-western parts of Styria (Wolf et al., 2017). Of the 10 strains from Austria in the current study, six were from Styria. In general, the strains from mountain pastures (Austria, Bavaria and Switzerland) showed higher strain diversity based on SNPs identified and also concerning CRISPR spacer array as compared to strains from plain regions (Lower Saxony, Mecklenburg-Western Pomerania, North Rhine-Westphalia and Schleswig-Holstein). This could also be attributed to the use of seasonal mountain pastures and the geology of the area where animals have more chance of being exposed to *C. chauvoei* spores and hence more pathogen-host interaction happens (Wolf et al., 2017).

The three strains isolated from the same host (animal) were observed with diversity representing non-synonymous substitutions. This hence indicates the possibility of emergence of polymorphic populations within a single host. Strains isolated from the same outbreak but different animals also showed SNP variations ranging from 12 to 68 and non-synonymous SNPs were dominating the variations (Table 1). The genes with variations showed very limited collinearity between outbreaks. Since the study involved the sequencing of only one colony from a clinical sample of the same animal or outbreak, the diversity of entire populations that occurs in the host during the infection is unclear. Although the study included very few strains that could be considered as targets for within-host evolution studies, the high number of unique, non-synonymous SNPs supports the speculation that strain diversification for the species could take place mainly in the host. The exact reason for strain diversity is unclear, as is the purifying selection in *Staphylococcus aureus* (Golubchik et al., 2013) or the involvement of recombination in *Helicobacter pylori*, where the initial inflammatory immune response during the acute phase of infection in the gastric epithelium causes a mutation burst in the bacteria (Linz et al., 2014).

The strains involved in studies of blackleg usually originate from outbreaks and the bacterium is believed to have evolved within the host where the anaerobic environment prevails. Hence all strains of this study have possibly undergone within-host evolution. Pangenome SNPs were mostly Non-synonymous (NS) indicating diversifying selection occurs in every strain. Also, because there is no evidence for a contagious nature of the pathogen, few reports suggest that the bacterium can have few replication cycles in the intestine before being either absorbed into the bloodstream or excreted (Abreu et al., 2017).

Since the study included isolates from different geographical locations and different outbreaks, each with limited genetic diversity, the strains were clustered based on pangenome SNPs using an alignment and reference-free method (Gardner et al., 2015). Pangenome SNPs were applied to cluster strains based on the presence of SNPs shared among the strains within a cluster (Figure 3). This phylogenetic tree and clusters indicated that repeated outbreaks at farm level can involve strains from the same lineages. The strains from Bavaria and Austria and Austria and Switzerland formed clusters which indicate that strains maintain relatedness within these geographical regions. The unique SNPs shared by a cluster were identified to have a maximum value of 76, which was obtained for the three strains from North Rhine-Westphalia. This was almost three times the number of SNPs defining unique clusters formed by strains from Germany. This indicates that the strains form a diverse cluster among the strains from Germany. The strains also showed uniqueness with respect to prophage composition and CRISPR spacer array. In contrast to most other outbreaks analyzed in this study, this outbreak was observed in a stable and not in a pasture. The bedding used to house the animals on the farm was made of cotton material, so the introduction of outbreak strains from other geographical origins may have been possible as well through fomites. North Rhine-Westphalia is considered as a region where blackleg is uncommon and no other outbreaks were reported at least since 1995 except for this unique outbreak.

No genetic variability for the CctA protein at the amino acid level was found for the strains involved in the current study. An earlier study has also reported CctA to be conserved except for one amino acid substitution for one strain each from New Zealand and United Kingdom (Rychener et al., 2017). Limited variability was observed for the NanA sialdiase and NagH, even though mostly variations were predicted for strains outside Germany, one strain from South Africa and unknown origins (Table 2). Antivirulence gene (AVG) expression in a pathogen is incompatible with the virulence of that pathogen. After acquiring a novel lifestyle by the pathogen, the antivirulence loci (AVL) are selectively inactivated either by point mutation, insertion, or deletion (Maurelli, 2007; Bliven and Maurelli, 2012). Interestingly, *nadA* and *nadB* genes reported as antivirulence loci in *Shigella* spp. (Prunier et al., 2007) along with *nadC* gene were found to be absent in all *C. chauvoei* genomes. On the other hand, the closely related species *C. septicum* showed the presence of an intact coding sequence for *nadA* whereas *nadB* and *nadC* genes were inactivated by point mutations. The requirement of NAD precursor by *Shigella* is met by converting exogenous nicotinic acid to nicotinic acid mononucleotide by nicotinate phosphoribosyltransferase (Prunier et al., 2007). Both *C. chauvoei* (12S0467: BTM20_07385) and *C. septicum* (DSM 7534: CP523_00230) genomes harbored nicotinate phosphoribosyltransferase [EC:6.3.4.21] gene. The presence/absence of potential antivirulence genes in genus *Clostridium* needs to be further explored in understanding the pathogenesis of blackleg.

Typing options for *C. chauvoei* strains involved in the current study were evaluated based on two approaches such as CRISPR spacer sequence matrix (Supplementary Figure 1) and cgMLST (Figure 4). The CRISPR spacer matrix diversity for the strains in the current study was found to be inadequate to differentiate many strains of regional/outbreak origins. Strains from Mecklenburg-Western Pomerania and most of the strains from Lower Saxony showed similar CRISPR spacer array. On the contrary, pangenome SNPs based phylogeny and cgMLST were able to differentiate these strains and harbored unique unshared SNPs. This hence proves the applicability of cgMLST in typing of strains from similar geographical origin and is of special value in investigating outbreaks. The CRISPR based typing approach can display genetic relatedness in a more general pattern. The sources of repeated outbreaks can be new strains with evolutionary relationship, which were freshly acquired by an animal causing the new outbreak or the rare probability that the herd carried the bacterium in latent stage and an outbreak was caused by any of the predisposing factors predicted for the pathogen at different time intervals. The usefulness of the limited cluster specific genes and SNPs to understand the evolution of this pathogen is obvious.

Studies have also pointed out the occurrence of blackleg outbreaks in vaccinated animals in Europe (Harwood et al., 2007; Wolf et al., 2017). Outbreaks with differing pathological findings from the classical form i.e., primary involvement of the tongue and intestine without skeletal muscle or heart involvement have also been reported (Harwood et al., 2007). Comparative genomics involving strains from classical blackleg (skeletal muscles) and visceral blackleg (heart, sublingual muscles and diaphragm) based on six strains showed highly conserved strain genomes present in both forms (Ziech et al., 2020). These reports could be suggestive of variant *C. chauvoei* strains present in the pastures which can evade vaccination and cause atypical outbreaks. The strain variants occurring within host, between host and even in repeated outbreaks at the same farm/pastures have to be investigated intensively in the future to understand strain evolution and pathogenesis of blackleg. The protein variants observed within these same host/outbreak strains point toward an entirely new array of targets that could be applicable in understanding the pathogenesis and immune evasion of the pathogen and may help to the develop novel vaccines. The difference in inactivation of potential AVL for *C. chauvoei* and *C. septicum* needs further evaluation to understand blackleg and malignant oedema.

## Data Availability Statement

The datasets presented in this study can be found in online repositories. The names of the repository/repositories and accession number(s) can be found in the article/Supplementary Material.

## Author Contributions

PT and CS conceptualized and designed the study. PT and MA-G performed the bioinformatics analysis. TS and IE performed next-generation sequencing. CS, HN, and LW supervised the study. PT, MA-G, and CS wrote the manuscript. CW, HN, CB, AB-D, WM, PZ, DG, DS, and US provided isolates and epidemiological data. All authors contributed to manuscript revision, read, and approved the submitted version.

## Conflict of Interest

The authors declare that the research was conducted in the absence of any commercial or financial relationships that could be construed as a potential conflict of interest.

## Publisher’s Note

All claims expressed in this article are solely those of the authors and do not necessarily represent those of their affiliated organizations, or those of the publisher, the editors and the reviewers. Any product that may be evaluated in this article, or claim that may be made by its manufacturer, is not guaranteed or endorsed by the publisher.

## Footnotes

1
https://www.fli.de

2
https://www.bioinformatics.babraham.ac.uk/projects/fastqc/

3
https://github.com/tseemann/shovill

4
https://github.com/tseemann/abricate

5
https://github.com/tseemann/snippy

6
https://github.com/MDU-PHL/pairwise_snp_differences

7
http://samtools.github.io/bcftools/bcftools.html

8
https://pubmlst.org/organisms/clostridium-chauvoei

9
https://github.com/tseemann/abricate

## Supplementary Material

Supplementary Figure 1 Schematic representation of CRIPR spacer array. CRISPR spacers identified in repeat regions one and two are represented as an array. The blank region between repeat regions one and two indicates the presence of a protein coding gene interrupting the spacer array. The orientations of spacers are represented as per DSM 7528^T^ strain and each unique spacer (45) is depicted with unique colored square plots. CRISPR spacers for all 64 strains were representing 18 unique patterns, each pattern was highlighted with own color. The region wise and country information are also represented. Most of the strains from Europe and the type strain had a similar CRISPR spacer composition. Schematic representation of CRISPR spacer array was generated using CRISPRStudio following prediction of CRISPR elements and spacers using CRISPRDetect program.

Supplementary Figure 2 BRIG BLAST atlas. BRIG blast atlas showing sequence variability among strains, with prophages and genomic islands from strain DSM 7528^T^ mapped to blast atlas. BLAST atlas was generated using BRIG software, with green; Austria, pink; Switzerland, dark green; Italy, purple; Canada, red; South Africa, gray; unknown. Arcs represent prophages (red) and genomic islands (blue) respectively. Genetic variation with respect to gene composition was observed among various strains within some prophages and genomic island predicted regions.

Supplementary Figure 3 Pairwise SNP divergence of strains based on geographical origin. Pairwise SNP divergence of strains based on geographical origin: The *Y*-axis depicts the number of SNP differences (median value); error bars indicate lower and upper inter-quartile (IQR) ranges and points represent the minimum and maximum values. **(A)** Germany vs Austria – Median pairwise SNP difference value of 89 (10 strains) for Austria as compared to 41 (38 strains) from Germany was observed. **(B)** Mountain vs Plain regions. The strains from the plain regions (Lower Saxony, Mecklenburg-Western Pomerania, North Rhine-Westphalia and Schleswig-Holstein) showed median pairwise SNP difference value of 38 (34 strains) where compared to 77 (17 strains) from mountain pastures (Bavaria, Austria, and Switzerland).

Supplementary Figure 4 Pie chart depicting the gene ontology classification of genes associated with host/outbreak strain genetic variability. Genes were annotated based on gene ontology terms (Blast2GO) for various biological processes. The number and percentage of genes involved in various biological process are shown in brackets. Genes involved in host/outbreak strain genetic variability were predominantly involved in cellular macromolecule and organo-nitrogen compound biosynthetic processes among others.

Supplementary Table 1 Details of *Clostridium chauvoei* strains involved in the study. Details of *Clostridium chauvoei* strains involved in this study showing number of strains, strain origin/sample number, Friedrich-Loeffler-Institut (FLI) strain designation, year, place and country of isolation. The NCBI Sequence Read Archive (SRA) and genome assembly accession numbers respectively for the strains are also depicted.

Supplementary Table 2 Genome assembly and annotation summary. The Table shows assembly (contig number, genome size, min, avg, max size of contig, and N50 values in bp) and annotation (CDS, rRNA, tRNA, and tmRNA) summary for the 64 strains involved in the study. Genome variability was very limited among the strains studied in terms of genome size and encoded proteins.

Supplementary Table 3 CRISPR spacers. The number of spacers identified for each strain at the repeat regions one and two are detailed in this Table. Unique spacer elements are numbered from 1 to 45 (spacers are numbered first with respect to DSM 7528^T^ (43 spacers) followed by other strains (2 spacers) whenever new spacers were observed). Spacer absences are represented by empty cells. Total number of spacers identified as well as separately for repeat regions one and two are also shown in the table. CRISPR elements and spacers were identified using the CRISPRDetect program.

Supplementary Table 4 Prophages and genomic islands. The identified prophages (1–4) and genomic islands (one to nine) are depicted in the table. Genomic coordinates as per DSM 7528^T^ strain for start and end positions and the size in bp are shown. The present genes are listed with number and product encoded. Prophages and genomic islands were predicted using Prophage Hunter tool and Island Viewer 4 respectively.

Supplementary Table 5 Pairwise SNP variations. Pairwise SNP variations among strains were deduced based on read mapping of the query strains with DSM 7528^T^ reference genome with snippy tool. Pairwise SNP variations ranged from 0 to a maximum of 331 SNPs between two isolates. The pairwise SNP variations among two isolates were higher when they were from different geographical regions.

Supplementary Table 6 SNP details of same host and outbreak strains. The list of SNPs identified in each strain (DSM 7528^T^ compared to outbreak strains), changes with respect to nucleotides and amino acids in case of non-synonymous substitutions (type of substitution), gene name and details of encoded proteins are shown in the table.
