# Supplementary figures and images for: Genome Sequence Analysis of Clostridium chauvoei Strains of European Origin and Evaluation of Typing Options for Outbreak Investigations
Source: Front Microbiol. 2021 Sep 29;12:732106. doi: 10.3389/fmicb.2021.732106 (PMC8513740; doi:10.3389/fmicb.2021.732106)

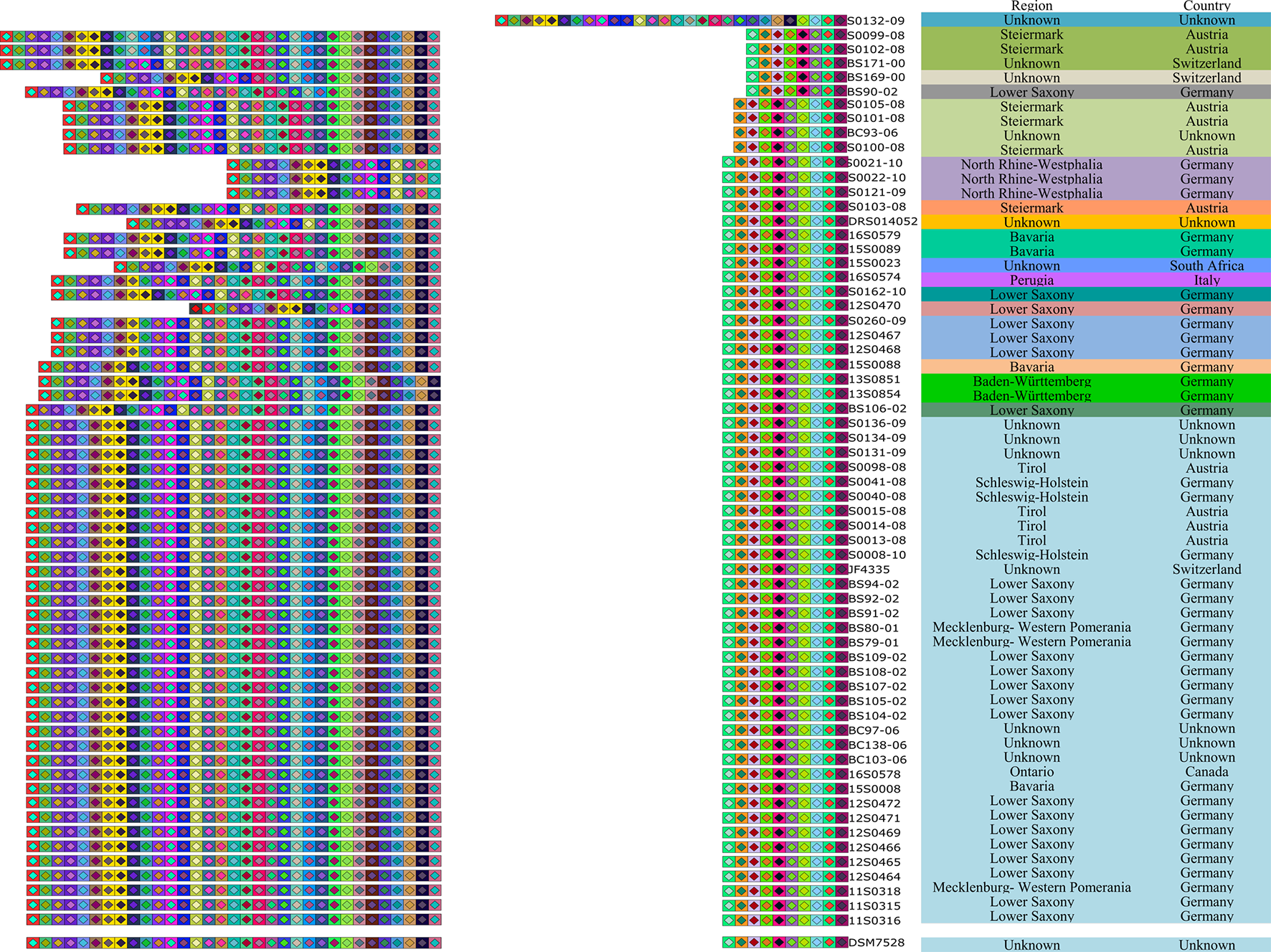

Supplement: Supplementary Figure 1 — Schematic representation of CRIPR spacer array. CRISPR spacers identified in repeat regions one and two are represented as an array. The blank region between repeat regions one and two indicates the presence of a protein coding gene interrupting the spacer array. The orientations of spacers are represented as per DSM 7528T strain and each unique spacer (45) is depicted with unique colored square plots. CRISPR spacers for all 64 strains were representing 18 unique patterns, each pattern was highlighted with own color. The region wise and country information are also represented. Most of the strains from Europe and the type strain had a similar CRISPR spacer composition. Schematic representation of CRISPR spacer array was generated using CRISPRStudio following prediction of CRISPR elements and spacers using CRISPRDetect program. [file Image_1.tif]

Figure S3A

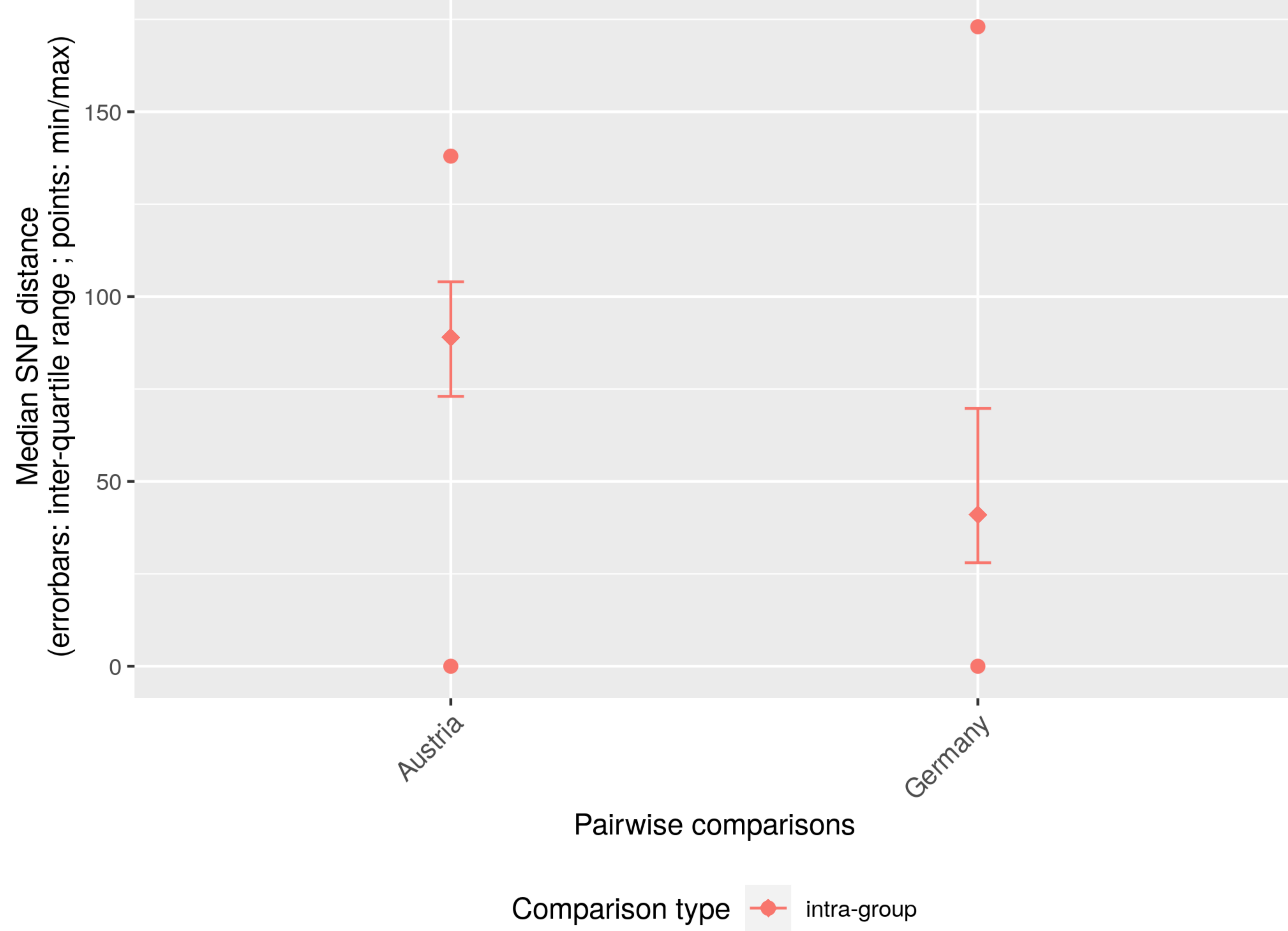

Figure S3B

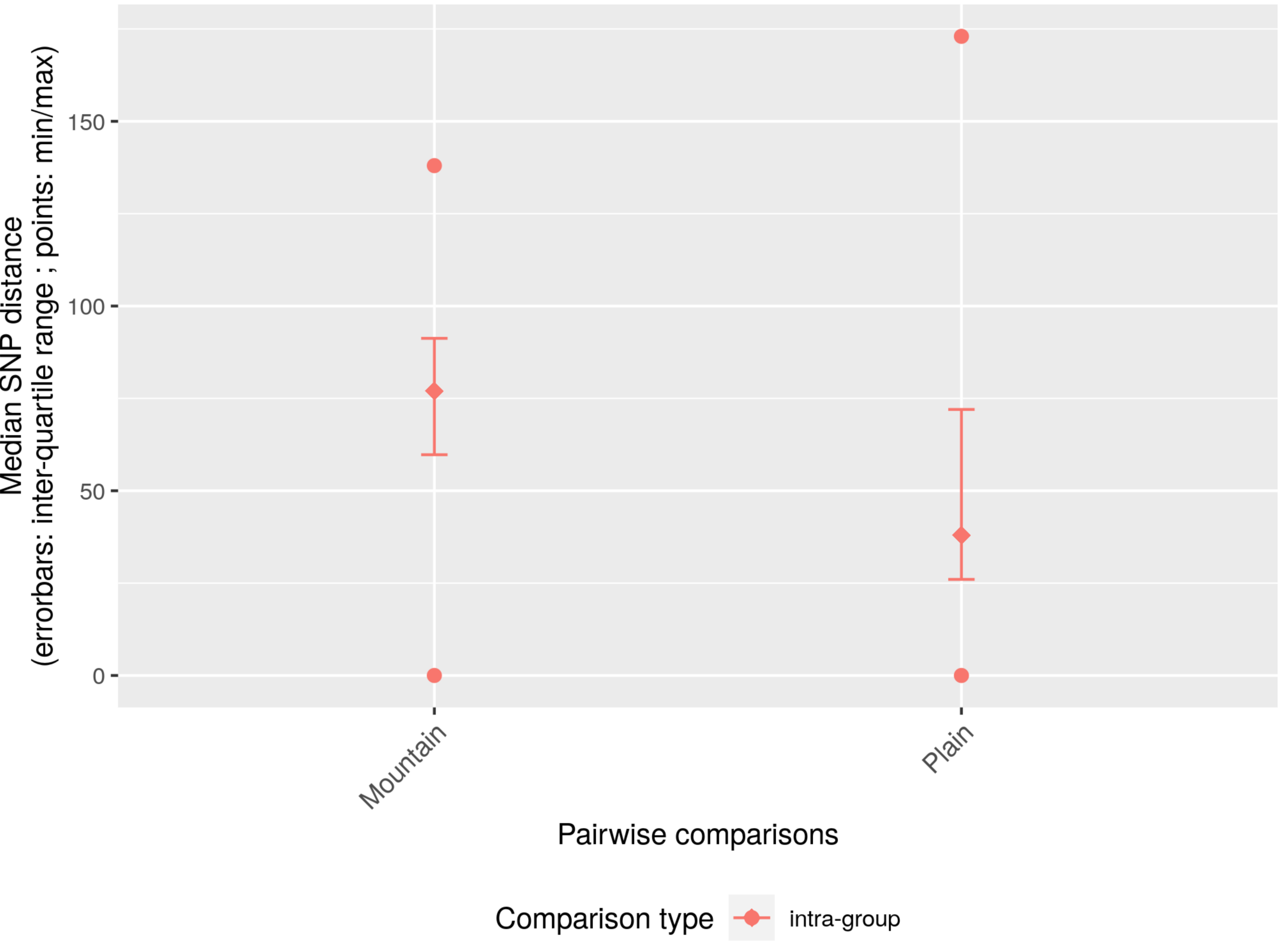

Supplement: Supplementary Figure 3 — Pairwise SNP divergence of strains based on geographical origin. Pairwise SNP divergence of strains based on geographical origin: The Y-axis depicts the number of SNP differences (median value); error bars indicate lower and upper inter-quartile (IQR) ranges and points represent the minimum and maximum values. (A) Germany vs Austria – Median pairwise SNP difference value of 89 (10 strains) for Austria as compared to 41 (38 strains) from Germany was observed. (B) Mountain vs Plain regions. The strains from the plain regions (Lower Saxony, Mecklenburg-Western Pomerania, North Rhine-Westphalia and Schleswig-Holstein) showed median pairwise SNP difference value of 38 (34 strains) where compared to 77 (17 strains) from mountain pastures (Bavaria, Austria, and Switzerland). [file Image_3.pdf]

# Sequence Distribution [Biological Process]

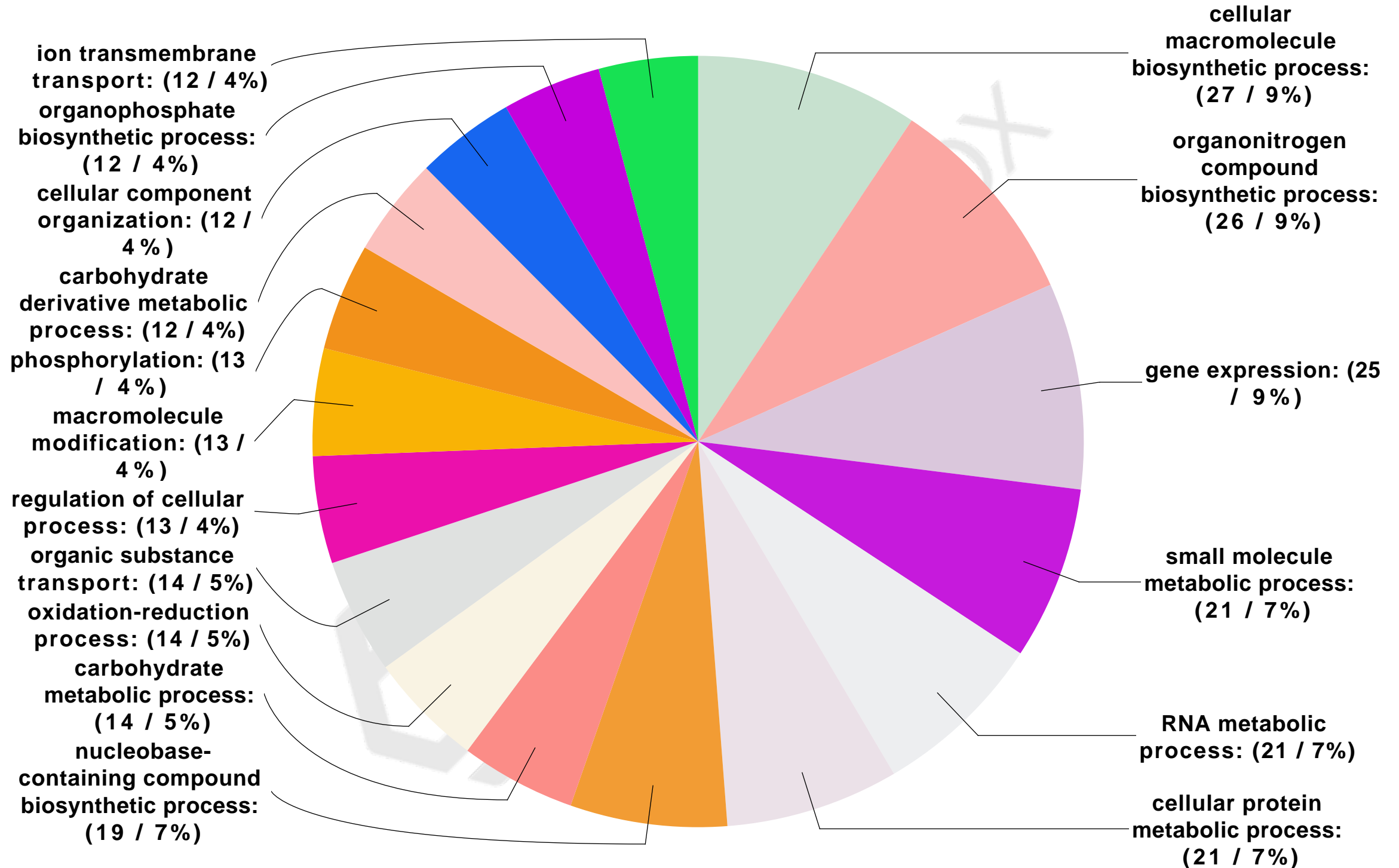

Supplement: Supplementary Figure 4 — Pie chart depicting the gene ontology classification of genes associated with host/outbreak strain genetic variability. Genes were annotated based on gene ontology terms (Blast2GO) for various biological processes. The number and percentage of genes involved in various biological process are shown in brackets. Genes involved in host/outbreak strain genetic variability were predominantly involved in cellular macromolecule and organo-nitrogen compound biosynthetic processes among others. [file Image_4.pdf]
