# Supplementary material for: Genome Sequence Analysis of Clostridium chauvoei Strains of European Origin and Evaluation of Typing Options for Outbreak Investigations
Source: Front Microbiol. 2021 Sep 29;12:732106. doi: 10.3389/fmicb.2021.732106 (PMC8513740; doi:10.3389/fmicb.2021.732106)
Supplement: Supplementary Figure 2 — BRIG BLAST atlas. BRIG blast atlas showing sequence variability among strains, with prophages and genomic islands from strain DSM 7528T mapped to blast atlas. BLAST atlas was generated using BRIG software, with green; Austria, pink; Switzerland, dark green; Italy, purple; Canada, red; South Africa, gray; unknown. Arcs represent prophages (red) and genomic islands (blue) respectively. Genetic variation with respect to gene composition was observed among various strains within some prophages and genomic island predicted regions. [file Image_2.pdf]

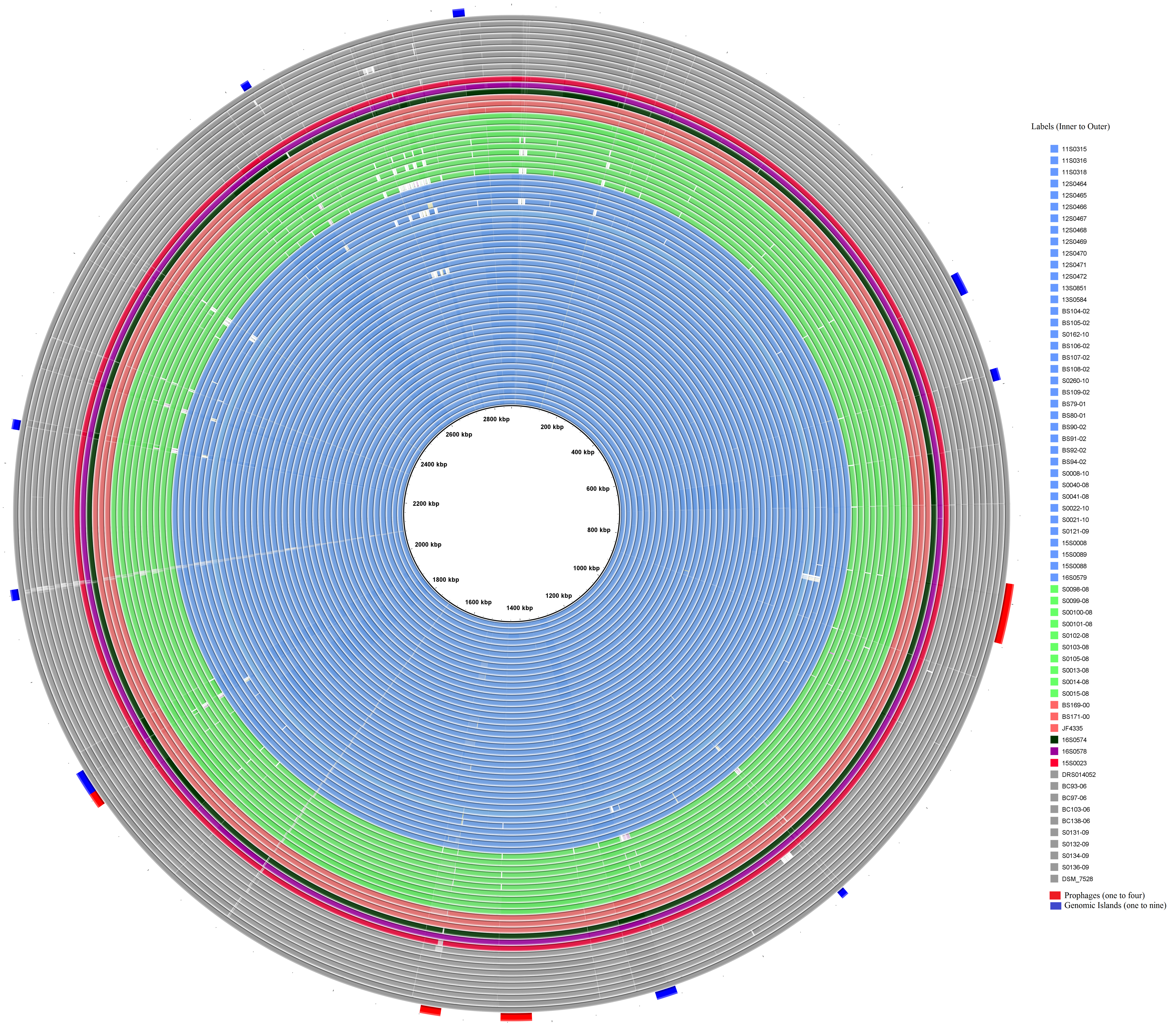
2800 kbp
200 kbp
400 kbp
600 kbp
800 kbp
1000 kbp
1200 kbp
1400 kbp
1600 kbp
1800 kbp
2000 kbp
2200 kbp
2400 kbp
2600 kbp
Labels (Inner to Outer)
11S0315
11S0316
11S0318
12S0464
12S0465
12S0466
12S0467
12S0468
12S0469
12S0470
12S0471
12S0472
13S0851
13S0584
BS104-02
BS105-02
S0162-10
BS106-02
BS107-02
BS108-02
S0260-10
BS109-02
BS79-01
BS80-01
BS90-02
BS91-02
BS92-02
BS94-02
S0008-10
S0040-08
S0041-08
S0022-10
S0021-10
S0121-09
15S0008
15S0089
15S0088
16S0579
S0098-08
S0099-08
S00100-08
S00101-08
S0102-08
S0103-08
S0105-08
S0013-08
S0014-08
S0015-08
BS169-00
BS171-00
JF4335
16S0574
16S0578
15S0023
DRS014052
BC93-06
BC97-06
BC103-06
BC138-06
S0131-09
S0132-09
S0134-09
S0136-09
DSM_7528
Prophages (one to four)
Genomic Islands (one to nine)
